# Supplementary material for: Vnn1 pantetheinase limits the Warburg effect and sarcoma growth by rescuing mitochondrial activity
Source: Life Sci Alliance. 2018 Jul 23;1(4):e201800073. doi: 10.26508/lsa.201800073 (PMC6238586; doi:10.26508/lsa.201800073)
Supplement: Supplementary file 1 [file LSA-2018-00073_TableS1.docx]

**Table S1: List of primers for qRT-PCR analysis**

| **Gene** | **Forward primer sequence** | **Reverse primer sequence** |
| --- | --- | --- |
| *Bnip3* | *TCCTGGGTAGAACTGCACTTC* | *GCTGGGCATCCAACAGTATTT* |
| *Pdk1* | *GGACTTCGGGTCAGTGAATGC* | *TCCTGAGAAGATTGTCGGGGA* |
| *Car9* | *TGCTCCAAGTGTCTGCTCAG* | *CAGGTGCATCCTCTTCACTGG* |
| *Hk2* | *TGATCGCCTGCTTATTCACGG* | *AACCGCCTAGAAATCTCCAGA* |
| *Glut1* | *CAGTTCGGCTATAACACTGGTG* | *GCCCCCGACAGAGAAGATG* |
| *Adm* | *CACCCTGATGTTATTGGGTTC* | *TTAGCGCCCACTTATTCCACT* |
| *Caveolin I* | *ATGTCTGGGGGCAAATACGTG* | *CGCGTCATACACTTGCTTCT* |
| *Collagen I* | *GCTCCTCTTAGGGGCCACT* | *CCACGTCTCACCATTGGGG* |
| *Actine* | TGGAATCCTGTGGCATCCATGAAACC | TAAAACGCAGCTCAGTAACAGTCCG |
